# N-3 Polyunsaturated Fatty Acids of Marine Origin and Multifocality in Human Breast Cancer

**DOI:** 10.1371/journal.pone.0147148

**Published:** 2016-01-26

**Authors:** Lobna Ouldamer, Caroline Goupille, Anne Vildé, Flavie Arbion, Gilles Body, Stephan Chevalier, Jean Philippe Cottier, Philippe Bougnoux

**Affiliations:** 1 Department of Gynecology, Centre Hospitalier Universitaire de Tours, Hôpital Bretonneau, 2 boulevard Tonnellé, 37044, Tours, France; 2 INSERM UMR1069, 10 boulevard Tonnellé, 37044, Tours, France; 3 François-Rabelais University, 10 boulevard Tonnellé, 37044, Tours, France; 4 Department of Radiology, Centre Hospitalier Régional Universitaire de Tours, Hôpital Bretonneau, 2 boulevard Tonnellé, 37044, Tours, France; 5 Department of Pathology, Centre Hospitalier Régional Universitaire de Tours, Hôpital Bretonneau, 2 boulevard Tonnellé, 37044, Tours, France; 6 INSERM UMR930, 10 boulevard Tonnellé, 37044, Tours, France; 7 Department of Oncology, Centre Hospitalier Régional Universitaire de Tours, Hôpital Bretonneau, 2 boulevard Tonnellé, 37044, Tours, France; INRA, FRANCE

## Abstract

**Objective:**

The microenvironment of breast epithelial tissue may contribute to the clinical expression of breast cancer. Breast epithelial tissue, whether healthy or tumoral, is directly in contact with fat cells, which in turn could influence tumor multifocality. In this pilot study we investigated whether the fatty acid composition of breast adipose tissue differed according to breast cancer focality.

**Methods:**

Twenty-three consecutive women presenting with non-metastatic breast cancer underwent breast-imaging procedures including Magnetic Resonance Imaging prior to treatment. Breast adipose tissue specimens were collected during breast surgery. We established a biochemical profile of adipose tissue fatty acids by gas chromatography. We assessed whether there were differences according to breast cancer focality.

**Results:**

We found that decreased levels in breast adipose tissue of docosahexaenoic and eicosapentaenoic acids, the two main polyunsaturated n-3 fatty acids of marine origin, were associated with multifocality.

**Discussion:**

These differences in lipid content may contribute to mechanisms through which peritumoral adipose tissue fuels breast cancer multifocality.

## Introduction

Epithelial breast tissue tumorigenesis may arise from a single or from several distinct sites within the same breast, leading to multifocality, defined as multiple simultaneous ipsilateral and synchronous breast carcinomas [1]. Such a cancer presentation appears to have a poor prognosis in breast cancer patients, with a greater probability of relapse and shorter survival than in women with unifocal tumors [2,3,4,5]. Mechanisms underlying multifocality remain unclear. The breast is an organ in which epithelial cells are embedded within a fat environment. The relationship between adipose tissue-derived lipid mediators and epithelial breast tissue biology has been thoroughly investigated and several molecular pathways related to the synthesis of lipid mediators have been described [6,7,8], giving rise to the hypothesis that the fatty acid profile of triglycerides stored in the adipose tissue might influence any malignant transformation of the mammary gland. Furthermore, the composition of adipose tissue fatty acids reflects past dietary habits, a widely recognized component of the risk and behavior of breast cancer [9,10,11,12], and several reports of circumstantial evidence support a potential role of adipose tissue in breast cancer development and aggressiveness. If multifocality does represent expression of the pressure of this immediate environment on the entire epithelial mammary tissue to generate growth of tumors at several sites in the same breast, then lipids surrounding this tissue in a breast with multifocal tumors might differ from that of a breast with a unifocal tumor. There is thus a need for study of the relationship between the fatty acid profile of breast adipose tissue and breast cancer multifocality.

We hypothesized that a specific pattern of composition of adipose tissue fatty acids may be associated with changes in cell functions which contribute to the onset of multifocal breast cancer. We studied the fatty acid profiles of samples of breast adipose tissue in a prospective cohort and investigated whether their composition differed according to tumor focality.

## Methods

The study was performed with approval of the Tours University Review Board. Written informed consent was obtained from all participants prior to the study.

The study cohort consisted of 23 consecutive female patients presenting with non-metastatic breast cancer proven by needle biopsy and scheduled for breast surgery at the University Hospital of Tours, France. All patients were Caucasian and from Central France. There were no lactating mothers. All patients underwent mammographic examination, directed breast ultrasonography and Magnetic Resonance Imaging (MRI) allowing assessment of breast tumor focality. The Radiologists (AV, JC) were dedicated breast radiologists accredited in breast radiology. All patients were examined with the same MRI protocol. All MR investigations were performed using a Siemens 3T MR scanner (a clinical 3T whole-body system (SiemensVerio, RF transmitter frequency = 123.22 MHz) and were performed in the prone position with a dedicated bilateral phased array breast coil (four-element two-channel coil, one channel per breast). One pathologist (FA), with experience in breast pathology and breast screening, reviewed the histology reports and biopsy slides and the surgical excision specimens. Invasive lesions were considered to be unifocal when only one invasive focus was observed on the large pathological sections, which may or may not have contained an in situ component. Multifocality was defined by the presence of multiple, well delineated invasive tumor foci separated from each other by uninvolved breast tissue comprising normal tissue, benign lesions or DCIS, regardless of the distance between foci.

### Sample details

Breast adipose tissue samples were obtained from the same consecutive 23 patients undergoing surgery at our hospital. Samples were excised from the external (tumor-free) region of the lumpectomy or mastectomy during surgery. Samples were stored in liquid nitogen to minimize degradation.

**Fig 1:**
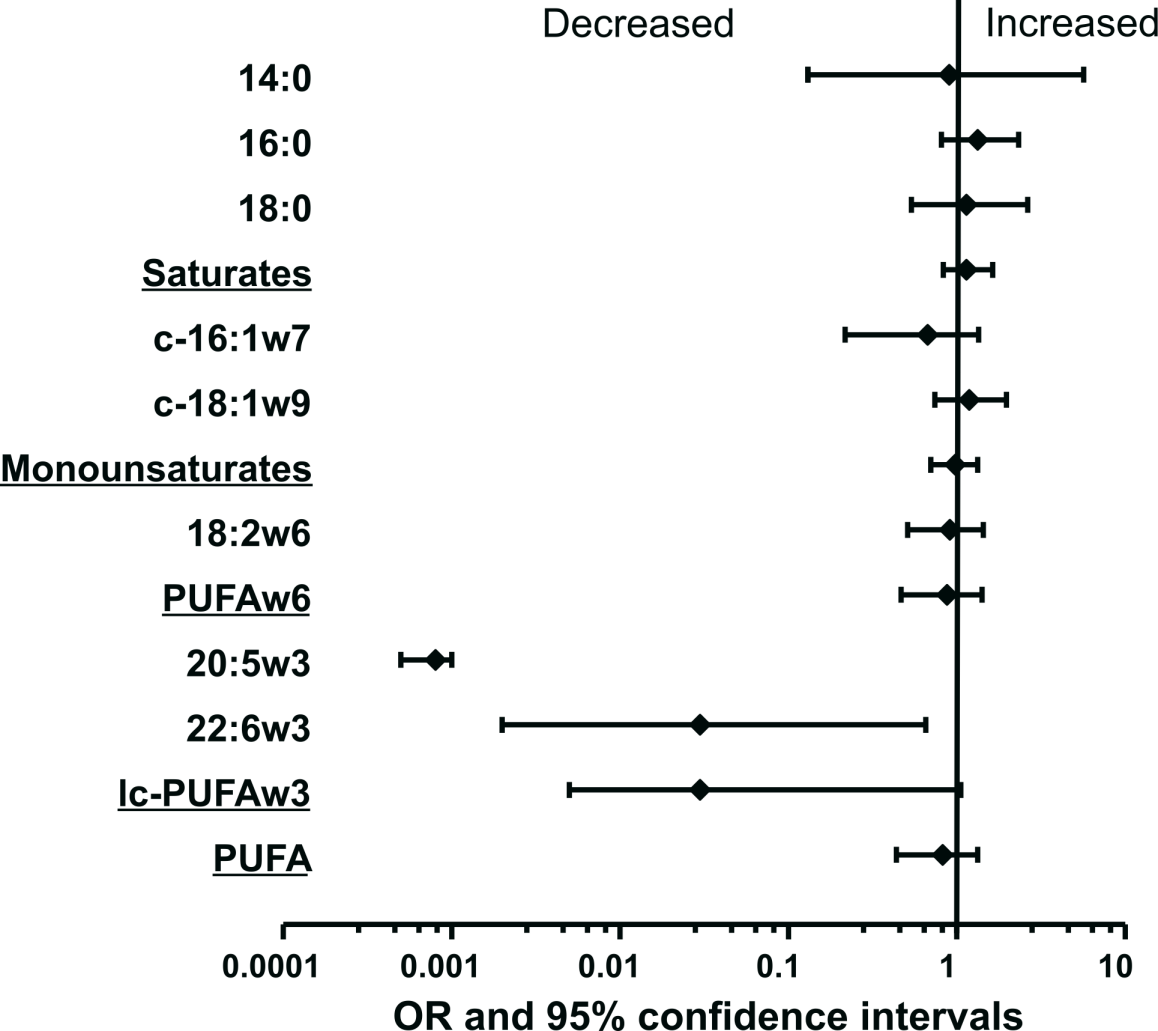
Forest plot of the association of multifocality and elevated level of fatty acid content in breast adipose tissue. Adipose tissue samples obtained during surgery from 23 patients with breast cancer were analyzed for fatty acid composition. X-axis shows relative increase in risk of multifocality expressed as odds ratio. Y-axis denotes different fatty acids. 14:0, 16:0 and 18:0 are the main saturated fatty acids, 16:1n-7, 18:1n-9 are the two main cis-monounsaturates,18:2 is the main n-6 polyunsaturate, PUFA n-6 include 18:2, 18:3 n-6, 20:2, 20:3, 20:4 and 22:6 n-6 polyunsaturates; LC-PUFAn3 include 20:5n3 and 22:6n-3, the two main long chain n-3 polyunsaturates, and 22:5 n-3, and PUFA is the sum of all individualized polyunsaturates. Analytic results are expressed with 95% confidence intervals.

To investigate whether there was any change in adipose tissue composition due to the presence of the breast tumor by itself, we collected a series of adipose tissue samples located at different distances from the tumor (0-10mm, 30-40mm, and >70mm on two orthogonal axes from seven additional patients scheduled to undergo a radical mastectomy for breast cancer. (Fig 1A)

### Lipid analysis

Total lipids were extracted from 50 mg frozen adipose tissue samples with chloroform methanol [13]. The lipid phase was evaporated under nitrogen gas. Triglycerides were purified by preparative Thin Layer Chromatography. Fatty acids were transmethylated with 14% boron trifluoride (BF3, Fluka, St. Quentin Fallavier, France). Fatty acid methyl esters (FAME) were dissolved in hexane and resolved by capillary gas chromatography on a GC-2010 Plus chromatograph (Schimadzu, France) equipped with an AOC20i autosampler (Schimadzu), an on-column injector and a flame ionization detector. A BPX70 column (60m x id 0.35mm, SGE, Courtaboeuf, France) was used for total FAME analysis with the column inlet pressure set at 130 kPA (constant pressure), hydrogen as carrier gas, and the following temperature program: 60°C (5min), 5°C/min 140°C (1min), 0.8°C/min 168°C (1 min), 0.3°C/min 175°C (10min) and 10°C/min 220°C (5min). FAME were identified by comparison of their retention times with those of authentic standards (Supelco, USA). Peak area was determined using GCSolution software (Shimadzu, France) and the results presented as percentage of total peak area. Peaks accounting for less than 1% of total area, such as alpha-linolenic acid (18:3 n-3) and docosahexaenoic acid (DHA, 22:6 n-3), were readily detected and quantified. Coefficients of variation (CV) were based on the analysis of one sample, aliquoted into 10 subsamples, all extracted and analyzed on the same day. CV ranged from 0.5% for the largest peaks to about 10% for the smallest peaks. The laboratory was blind to links between samples and clinical data.

### Statistical analysis

Statistical analyses were performed with R2.13.1 (http://www.cran.r-project.org/) software and GraphPad Prism 5 software (GraphPad Software, Inc, san Diego, USA). Demographic and baseline clinical characteristics were summarized using means± standard deviation (SD) for continuous variables and percentages for categorical variables. The Mann-Withney test or Fischer’s exact test were used to compare categorical values. The Wilcoxon test was used for continuous values (to compare levels of fatty acids between unifocal and multifocal groups). We considered p≤0.05 to be statistically significant.

## Results

The characteristics of the population according to focality are described in Table 1. Five of the 23 patients (21.7%) had multifocal tumors. There were no differences in age or BMI between the two groups. Eight patients in the unifocal group had normal BMI, three were obese and seven were overweight. In the multifocal group, two patients had normal BMI, two were overweight and one obese. Levels of the main fatty acids stored in breast adipose tissue are presented in Table 2, according to focality. Significant differences were found between the fatty acid profiles of adipose tissue of patients with unifocal tumors and those of patients with multifocal tumors. Eicosapentaenoic acid (EPA), docosahexaenoic acid (DHA) and long chain n-3 PUFA levels were higher in patients with unifocal tumors than in patients with multifocal tumors (p = 0.01, p = 0.01 and p = 0.01, respectively). There were no significant differences with respect to other FA: linoleic acid (LA, the essential n-6 PUFA), arachidonic and alpha linolenic acids were equally distributed (p = 0.59, p = 0.54, p = 0.29, respectively). Relative risk of multifocality by fatty acid is presented in Fig 1. Odds ratios were significantly low for the two long chain polyunsaturated n-3 fatty acids EPA and DHA.

**Table 1:** Patient and tumor characteristics.

Characteristics		Unifocal (n = 18)	Multifocal (n = 5)	p
Age, y	mean [SD]	56.4 ±10.38 [36–70]	64.0 ±15.57 [46–87]	0.35
BMI, kg/m^2^	mean [SD]	27.46 ± 6.96 [20.5–47.9]	26.38 ± 3.88 [20.5–30]	0.66
Menopause	n (%)	11(61.1)	3 (60)	1
Personal history of breast cancer	n (%)	4 (22.2)	0	0.009
Familial history of breast cancer	n (%)	6 (33.3)	3 (60)	0.34
Radiological size, mm	mean [range]	23.1 ± 18.07 [6–80]	31.2 ± 17.52 [20–60]	0.39
Histological size of invasive component, mm	mean [range]	24.4 ± 21.55 [6–90]	34.0 ± 21.48 [15–67]	0.41

BMI, body mass index

**Table 2:** Gas chromatography assessment of fatty acid composition of adipose tissue.

Fatty acids	Unifocal (Mean* ± SD [range])	Multifocal (Mean* ± SD [range])	p
14:0	3.19 ± 0.60 [2.38–4.25]	3.17 ± 0.41 [2.59–3.69]	0.90
16:0	22.67 ± 2.17 [20.42–28.05]	23.81 ± 0.89 [22.53–25.05]	0.09
18:0	4.80 ± 1.39 [1.98–6.82]	5.03 ± 1.32 [3.62–7.18]	0.74
Total SFA	31.50 ± 3.55 [25.5–37.72]	32.82 ± 1.84 [30.82–35.63]	0.28
16:1	4.37 ± 1.86 [1.89–9.67]	3.63 ± 0.65 [2.67–4.25]	0.17
18:1 n-9c	43.86 ± 2.32 [40.21–47.64]	44.64 ± 1.71 [42.4–47.13]	0.43
18:1 n-7c	2.43 ± 0.57 [1.64–3.72]	2.30 ± 0.27 [1.83–2.53]	0.48
Total MUFA	52.50 ± 3.63 [46.45–59.83]	52.25 ± 2.17 [48.54–54.28]	0.86
18:2 n-6c	10.25 ± 2.45 [5.67–16.92]	9.84 ± 1.06 [8.80–11.00]	0.59
20:4 n-6	0.40 ± 0.11 [0.25–0.60]	0.35 ± 0.13 [0.27–0.58]	0.54
Total n-6 PUFA	11.64 ± 2.38 [7.55–17.96]	11.13 ± 0.94 [9.99–12.13]	0.47
18:3 n-3c	0.63 ± 0.16 [0.32–0.92]	0.56 ± 0.12 [0.39–0.70]	0.29
20:5 n-3	0.07 ± 0.04 [0.02–0.15]	0.04 ± 0.02 [0.01–0.06]	**0.01**
22:5 n-3	0.25 ± 0.09 [0.10–0.36]	0.20 ± 0.04 [0.10–0.27]	0.16
22:6 n-3	0.20 ± 0.09 [0.04–0.35]	0.12 ± 0.05 [0.04–0.16]	**0.017**
Total n-3 PUFA	1.20 ± 0.25 [0.79–1.72]	0.95 ± 0.13 [0.82–1.14]	**0.01**

* expressed as % area

**Fig 2:**
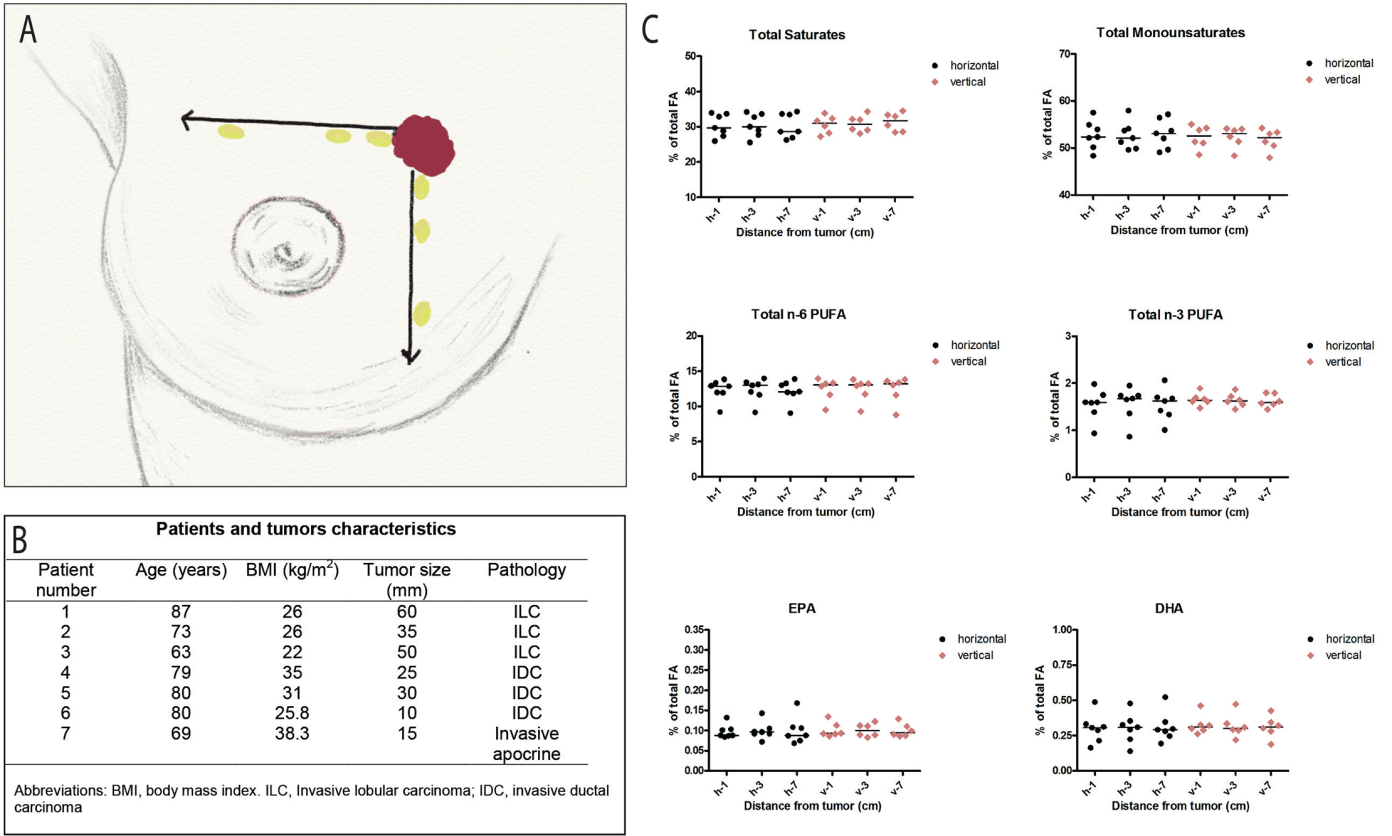
Fatty acid composition of breast adipose tissue according to distance from the tumor. A) Three breast adipose tissue specimens obtained from seven consecutive patients who underwent radical mastectomy for invasive breast carcinoma were sampled at different distances from the tumor (0-10mm, 30-40mm, and >70mm) in two orthogonal axes. Lipids were extracted and fatty acids analyzed as described in Materials and Methods. B) Characteristics of the seven patients and their tumors. C) Fatty acid levels of the seven patients according to distance from the tumor on the horizontal axis (black symbols) and vertical axis of the breast (red symbols). EachsSymbols represent values from one patient, expressed as % area of total fatty acids; lines, median value. From upper left to lower right: total saturates, total monounsaturates, total n-6 PUFA, total n-3 PUFA, EPA, DHA. There was no significant variation in fatty acid levels according to site of sampling for any patient.

To ensure that the differences in the adipose tissue composition were not simply a consequence of the distance of breast adipose tissue samples from the breast tumor, we sampled adipose tissue at different distances from the tumor in a separate population of seven consecutive patients undergoing radical mastectomy. There were no differences in the fatty acid profiles of any samples for any patient, whatever the distance or the axis of the site of sampling within the same breast (Fig 2).

## Discussion

This pilot study of 23 women consecutively treated for non-metastatic breast cancer was undertaken to evaluate whether possible differences in breast adipose tissue lipid composition might be associated with breast cancer multifocality. We found an association between low levels of DHA and EPA in breast adipose tissue and multifocality in breast carcinoma.

The close proximity of the adipose tissue fragments analyzed to the primary tumor raised the question of a possible reciprocal effect of the tumor on the levels of these fatty acids in adipose tissue. The lack of variation in fatty acid levels according to the distance of the adipose tissue sample from the tumor definitively indicated that the tumor does not itself influence the lipid environment, at least for DHA and EPA. This is in line with findings previously obtained from an experiment carried out in rats with mammary tumors, where we reported a lack of association between tumor burden as a consequence of tumor growth and the evolution of the alpha linolenic acid level in the adipose tissue [14]. Although the metabolism of alpha linolenic acid, the essential fatty acid precursor of the long chain n-3 PUFA EPA and DHA, differs, the probability of a direct effect of the tumor on n-3 PUFAs levels in human breast adipose tissue is therefore very low.

The significance of this association is not currently known. If multifocality represents expression of an influence of the immediate environment on the mammary gland epithelial tissue to make mammary tumors grow at several sites in the same breast, and assuming that long chain n-3 fatty acids oppose the development of tumors in experimental mammary tumor models, it could be speculated that low levels of long chain n-3 PUFAs permit tumor development at multiple sites within a same breast epithelial tissue and therefore authorize multifocality. The lack of an experimental model of multifocality makes this hypothesis difficult to investigate. An association between the level of n-3 PUFA in adipose breast tissue and the prognosis of breast cancer has already been reported [15]. Since multifocality is linked to breast cancer prognosis, it is not surprising to find a similar association between n-3 fatty acid levels and multifocality.

The origin and presentation of breast cancer are multifactorial and involve genetic, hormonal, environmental and nutritional factors [16]. The lipid composition of adipose tissues has been considered to be the best qualitative biomarker of past dietary intake of fatty acids [9,10,11,12]. The levels of DHA and EPA in this storage tissue may therefore reflect past intake of long chain FA of marine origin. This suggests the possibility of a link between dietary habits and the clinical expression of breast cancer [6,17,18,19].

Many mechanisms have been proposed to account for the effects of n-3 PUFAs on cancer [8]. These include changes in toxicity to stem cells [20], metabolic programing and morphological development of the mammary gland [21], cell proliferation [22,23], production of inflammation-inducible cytokines and eicosanoids [24], PPAR activation [25,26], fatty acid synthesis and metabolism [27,28], angiogenesis [29,30], and lipid peroxidation [31] to list but a few.

In summary, this pilot study revealed key biochemical differences in the adipose tissue taken from women with multifocal breast tumors compared to that from unifocal tumors. Despite only examining a small number of patients, there was a significant difference in EPA and DHA levels between uni- and multifocal breast tumors, suggesting that the difference observed is authentic. However, additional patient samples are needed to determine the validity of the correlations observed in this pilot study.
